# Supplementary material for: Resolved magnetohydrodynamic wave lensing in the solar corona
Source: Nat Commun. 2024 Apr 16;15:3281. doi: 10.1038/s41467-024-46846-z (PMC11021502; doi:10.1038/s41467-024-46846-z)
Supplement: Supplementary file 1 — Description of Additional Supplementary Files [file 41467_2024_46846_MOESM1_ESM.docx]

**Description of Additional Supplementary Files**

**File Name: Supplementary Movie I
Description:** 193 Å movie of evolution of the wave with the duration of about 1 hour observed by SDO/AIA on 2011 February 24.

**File Name: Supplementary Movie 2
Description:** Evolution of the wave in a Numerical simulation based on the coronal hole profile observed by STEREO-B/EUVI.

**File Name: Supplementary Movie 3**

**Description:** Evolution of the wave in an idealized numerical simulation.
