# Supplementary material for: Resolved magnetohydrodynamic wave lensing in the solar corona
Source: Nat Commun. 2024 Apr 16;15:3281. doi: 10.1038/s41467-024-46846-z (PMC11021502; doi:10.1038/s41467-024-46846-z)
Supplement: Supplementary file 3 — Source Data [file 41467_2024_46846_MOESM3_ESM.zip › resupply source data/Supplementary information.docx]

**Source DATA information**

The description is placed in the folder corresponding to each source data.
